# Velocity and Out-Step Frequencies for a Micro-Swimmer Based on Spiral Carbon Nanotubes

**DOI:** 10.3390/mi14071320

**Published:** 2023-06-27

**Authors:** Ce Zhang, Shiqi Ma, Lizhong Xu

**Affiliations:** School of Mechanical Engineering, Yanshan University, Qinhuangdao 066000, China; zhangce@stumail.ysu.edu.cn (C.Z.); mashiqi@stumail.ysu.edu.cn (S.M.)

**Keywords:** micro-swimmers, spiral carbon nanotubes, velocity, out-step frequency

## Abstract

The existing producing processes of micro spiral swimmers are complex. Here, a microswimmer with a magnetic layer on the surface of the spiral carbon nanotubes is proposed, which has a simple producing process. For the microswimmer, its equations of the velocities and out-step frequency are deduced. Using these equations, the velocities and out-step frequency of the microswimmer and their changes with related parameters are investigated. Results show that its velocities are proportional to the radius and helix angle of the spiral carbon nanotubes, and its out-step frequencies are proportional to magnetic field strength, the helix angle and magnetic layer thicknesses of the spiral carbon nanotubes, and inversely proportional to the fluid viscosity. The out-step frequency of the microswimmer is measured, which is in good agreement with the calculative ones.

## 1. Introduction

Microswimmers can perform extremely delicate tasks in very small spaces and have broad application prospects in fields such as biomedicine, etc. [1,2]. Microswimmers can perform targeted drug transportation by injecting into the human body, delivering drugs to the lesion site, and releasing them for targeted treatment [3,4,5]. In minimally invasive surgery, microswimmers can operate in the eyes, inner ear, and cardiovascular system [6,7].

The main driving methods for microswimmers include electrostatic field drive [8,9], external magnetic drive [10,11], ultrasonic drive [1], and optical drive [12,13]. Among them, the external magnetic field drive has a fast response and precise control. Aside from those, magnetic fields can penetrate the human body, and low-strength magnetic fields are harmless to the human body, making their application very convenient.

Magnetic drives are mainly divided into the magnetic gradient drive and the spiral propulsion drive. In microfluidics, the magnetic gradient drive requires greater magnetic field strength, and spiral propulsion can generate greater propulsion force [14].

In 1996, Honda first proposed a spiral magnetic microswimmer, which consists of a square magnetic head with a side length of 1 mm and a spiral structure with a diameter of 1 mm and a length of 21.7 mm. The external magnetic field drives the magnetic head to rotate, while the magnetic head drives the spiral structure at the tail to rotate [15]. So far, spiral magnetic microswimmer technology has gained widespread attention and in-depth research.

Tottori proposed a six-finger spiral magnetic microswimmer, where the micro gripper at the head is composed of six rigid fingers. The spiral magnetic microswimmer has a diameter of 1 μm–8 μm, and the experiment of grasping polystyrene particles in pure water was completed [16]. Peyer designed an artificial flagellum swimmer, which is similar in size to *Escherichia coli* and consists of a square magnetic plate head and a spiral tail [17]. Gao separated the spiral woody vessel fibers from the leaves of vascular plants which are of a regular spiral shape, and coated a magnetic film on the separated spiral structure, which can be driven and propelled in a magnetic field [18,19].

Lee applied the spiral magnetic swimmer to unblock blood clots at lesions in the vascular network. After the microspiral magnetic swimmer entered the human body, the external magnetic field was adjusted to accurately guide it to the lesion in the vascular network, which started to drill through the thrombus, thus opening the way for blood circulation [20]. Islam used a Hall effect sensor array and pre-calculated magnetic field maps of two synchronous rotating dipole fields to estimate the position of the spiral swimmer; the closed-loop motion control of a spiral swimmer was achieved using the rotating dipole field, and the positioning accuracy of this method was verified through visual feedback and feature tracking within the in vitro model [21]. Leclerc studied a micromagnetic swimmer and a control device in which the swimmer has a diameter of 2.5 mm and a length of 6 mm. It contains an internal permanent magnet driven by the rotating magnetic field of the external magnetic system and can perform three-dimensional path tracking and blood clot removal [22]. Lee proposed a new type of intravascular spiral magnetic microswimmer, which completed spiral navigation, dredging, object transfer, and various functional motion experiments in a tubular environment simulating human blood vessels [23]. Sa studied a spiral magnetic microswimmer with a serrated head and optimized the pitch length of the serrated head, verifying the superiority of the serrated head over the cone-shaped head by experiments [24]. Kadiri developed a biocompatible iron platinum (FePt) alloy for driving helical microswimmers in low Reynolds number environments. FePt coating has catalytic activity and can be used for Janus microswimming devices controlled by both light and magnetic fields [25]. Yan designed a cell-based magnetic microswimmer using multicellular spirulina and controlled the release rate of molecules through the thickness of magnetite coating. This technology can provide a feasible method for the gastrointestinal delivery of molecular agents [26]. Liu introduced a microswimmer made by coating superparamagnetic Fe_3_O_4_ nanoparticles onto Chlorella microalgae, which has magnetic driving and navigation capabilities. Under the guidance of the rotating magnetic field, the microswimmer can accurately move to the target muscle bundle and can accurately activate the muscle at the micro level [27].

In summary, magnetic micro spiral swimmers have undergone rapid development, and various new manufacturing processes for magnetic micro spiral swimmers have emerged which include self-scrolling [28], direct laser writing [29], glancing angle deposition [30,31], template-assisted electrodeposition [32], and biotemplating synthesis [33]. Using these processing technologies, microswimmers with different structures and sizes have been developed for different working environments. However, the existing machining processes are complex and expensive, which hinders the advances and application of microswimmer technology.

In this paper, a microswimmer with a magnetic layer on the surface of the spiral carbon nanotubes is proposed. For the microswimmer, its mechanics model is proposed, and the equations of the velocities and out-step frequency are deduced. Using these equations, the velocities and out-step frequency of the microswimmers and their changes with related parameters are investigated. The microswimmers are produced and measured. The experimental results are compared with the calculative ones. 

## 2. Structure Composition and Operation Principle

Micro spiral structures are complicated and expensive to process. Here, low-cost spiral carbon nanotubes are selected as the skeleton of the micro-swimmers, and a magnetic thin film is steamed on the spiral carbon nanotube by evaporation coating technology to produce the magnetically driven micro spiral swimmers.

**Figure 1:**
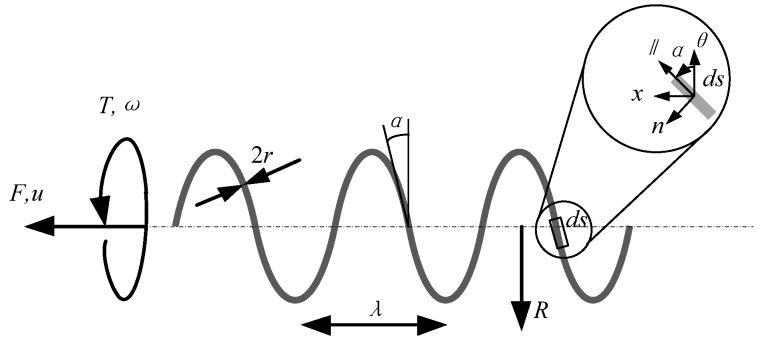
Geometric parameters of the microswimmer.

The geometric parameters of the microswimmer are shown in Figure 1. It is mainly composed of an internal multi-wall spiral carbon nanotube and a magnetic layer on the surface. *r* is the radius of slender wire, λ is the pitch of a helix, *F* is the non-fluid force on the microswimmer, *T* is the non-fluid torque on the microswimmer, *R* is the helix radius, α is the helix angle, and *u* is the velocity of the microswimmer.

The micro-swimmer is placed in a uniform magnetic field without gradient, and the magnetic layer of the micro-swimmer is magnetized. When the magnetic field rotates, the magnetic layer of the micro-swimmer is affected to generate a torque of rotation around the center axis of the micro-swimmer. During rotation, the spiral structure interacts with the surrounding fluid to produce thrust along the axis of the micro-swimmer. Under the action of axial thrust, a micro-swimmer can generate forward or backward motions.

## 3. Velocity and Out-Step Frequency of the Microswimmer

The basic principle of the magnetic drive is to apply magnetic force or magnetic torque to magnetized micro-objects. Among them, the magnetic field force *F*_m_ is affected by the magnetic field gradient, and the magnetic moment *T*_m_ is influenced by the strength of the magnetic field.

A magnetized object will receive a thrust and a torque in a magnetic field. The formula for the magnetic force and torque is

(1) $$T_{m}=VM\times B$$

(2) $$F_{m}=V\left(M\nabla \right)B$$

where M is the magnetization strength, ***B*** is the magnetic induction intensity, and *V* is the volume of the magnetized object.

Here, metal nickel is selected as the magnetic material, nickel is a ferromagnetic material, saturation magnetization *M* is a constant, and magnetic induction intensity can be controlled by changing the current in the coils.

When the magnetization direction of the magnetic layer of the microswimmer is collinear with the magnetic field direction, and the torque ***T****_m_* subjected to the microswimmer is zero, the microswimmer will not move. When the magnetization direction of the magnetic layer of the microswimmer is perpendicular to the magnetic field direction, the torque ***T****_m_* subjected to the microswimmer is the maximum, and the velocity of the microswimmer will be the maximum. 

When a microswimmer moves in a fluid, in addition to the influence of a magnetic field, it is also affected by fluid resistance. In 1976, Lighthill proposed the resistance theory to calculate the resistance of micro helical structures in fluids [34]. The resistance coefficient is

(3) $$\left\{\begin{array}{c} \xi_{∥}=\frac{2\pi \eta }{\ln\left(2q/r\right)-0.5}\\ \xi_{n}=\frac{4\pi \eta }{\ln(2q/r)+0.5} \end{array}\right.$$

where $\xi_{∥}$ is the resistance coefficient parallel to the cylindrical axis, ξ*_n_* is the rresistance coefficient perpendicular to the cylindrical axis, *q* = 0.09*λ*. 

For the steady-state motion of a microswimmer, the externally applied forces and moments must be equal to the resistance the microswimmer experiences in the fluid. Thus, the relationship between the forces/moments and velocity/frequency could be given by

(4) $$\left[\begin{array}{c} F\\ T \end{array}\right]=\left[\begin{array}{cc} a & b\\ b & c \end{array}\right]\left[\begin{array}{c} u\\ \omega \end{array}\right]$$

where a,b,*c* are the coefficients related to the geometric parameters of the microswimmer and fluid viscosity. 

The coefficients a,b,*c* could be given from the resistance theory:

(5) $$a=2\pi nR\left(\frac{\xi_{∥}\cos^{2}\left(\alpha \right)+\xi_{n}\sin^{2}\left(\alpha \right)}{\sin\left(\alpha \right)}\right)$$

(6) $$b=2\pi nR^{2}\left(\xi_{∥}-\xi_{n}\right)\cos\left(\alpha \right)$$

(7) $$c=2\pi nR^{3}\left(\frac{{\xi_{∥}\sin}^{2}\left(\alpha \right)+\xi_{n}\cos^{2}\left(\alpha \right)}{\sin\left(\alpha \right)}\right)$$

where n is the helix turn number.

Since the entire body of the microswimmer is helical and the magnetic field is uniform, the external applied force could be considered to be equal to zero [18], that is, *F* = 0. Thus, Equation (4) can be changed into following form:

(8) $$\left[\begin{array}{c} 0\\ T \end{array}\right]=\left[\begin{array}{cc} a & b\\ b & c \end{array}\right]\left[\begin{array}{c} u\\ \omega \end{array}\right]$$

The velocity *u* of the microswimmer as a function of the rotating magnetic field angle frequency ω can be given as:

(9) $$u=-\frac{b}{a}\omega$$

Substituting Equations (5) and (6) into (9), yields

(10) $$u=-\frac{R\left(\xi_{∥}-\xi_{n}\right)\cos\left(\alpha \right)\sin\left(\alpha \right)}{\xi_{∥}\cos^{2}\left(\alpha \right)+\xi_{n}\sin^{2}\left(\alpha \right)}\omega$$

Equation (10) shows that the velocity *u* of the microswimmer depends on the rotating magnetic field angle frequency ω, the radius *R*, the pitch λ of a helix, helix angle α, the radius *r* of cabon nanotube wire, independent of fluid viscosity. 

Equation (10) also shows that the velocity of the microswimmer is proportional to the rotational angular frequency of the magnetic field, that is, the faster the magnetic field frequency, the faster the velocity of the microswimmer. However, there is a maximum value for the velocity of the microswimmer, and after exceeding the maximum value, the speed will quickly decrease with an increase of the magnetic field frequency. The magnetic field frequency corresponding to the maximum velocity is called the out-step frequency.

The reason for this is that as the velocity of the microswimmer increases, the resistance it receives in the fluid becomes greater and greater. When the resistance torque is equal to the maximum magnetic torque on the microswimmer, the microswimmer reaches the out-step frequency, and the velocity of the microswimmer reaches its maximum value.

As the rotational angular frequency of the magnetic field continues to increase, the magnetic torque on the microswimmer is insufficient to maintain the same rotational speed as the magnetic field, resulting in the microswimmer entering the out-step state.

Combining Equations (8) with (9), the out-step frequency of the microswimmer can be given by:

(11) $$\omega_{max}=\frac{a}{ac-b^{2}}T_{m,max}$$

Substituting Equations (5)–(7) into (11), yields

(12) $$\omega_{max}=\frac{\frac{\xi_{∥}\cos^{2}\left(\alpha \right)+\xi_{n}\sin^{2}\left(\alpha \right)}{2\mathrm{\pi n}R^{3}\sin\left(\alpha \right)}T_{m,max}}{\left(\frac{{(\xi }_{∥}\cos^{2}\left(\alpha \right)+\xi_{n}\sin^{2}\left(\alpha \right))({\xi_{∥}\sin}^{2}\left(\alpha \right)+\xi_{n}\cos^{2}\left(\alpha \right))}{\sin^{2}\left(\alpha \right)}-{\left(\xi_{∥}-\xi_{n}\right)}^{2}\cos^{2}\left(\alpha \right)\right)}$$

From Equation (1), it can be deduced that the maximum torque *T_m,max_* is related to the magnetization strength, outer magnetic field intensity, and the volume of the magnetized object.

Because the magnetic material is given, the saturated magnetization of nickel is a fixed value, but the magnetic field strength and the thickness of the magnetic layer are adjustable parameters.

Thus, the driving magnetic field frequency range of microswimmers can be designed. The out-step frequency of the microswimmer can be changed by changing the magnetic field intensity and the thickness of the magnetic layer. Here, the magnetic field intensity is the most effective control factor.

## 4. Results and Discussion

**Figure 2:**
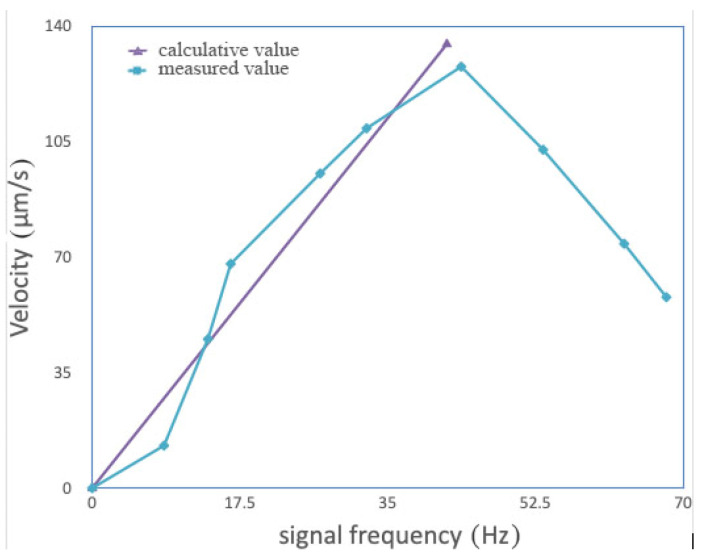
Velocity changes of the microswimmers along with signal frequency.

Using the above equations, the velocities of the microswimmers and their changes, along with the signal frequency are investigated. Here, the wire radius r of the spiral carbon nanotube is 0.2 μm, its helix pitch λ is 1μm, the frequency of the driving magnetic field is 40 Hz, the thickness of the magnetic layer is 80 nm, the magnetic field intensity is 8 mT, the fluid viscosity η is 2.5 mPa·s, the radius *R* = 1.4 μm, and the helix angle α = 5°. The velocities of the microswimmers are calculated and measured (see Figure 2). 

When the signal frequency is below some values, the velocity of the microswimmer is proportional to the signal frequency. Here, the calculated values are in good agreement with the measured results. When the signal frequency is equal to 43.7 Hz, the velocity of the microswimmer reaches its maximum, 127.8 μm/s. When the signal frequency is above 43.7 Hz, the velocity of the microswimmer decreases rapidly with increasing signal frequency. The signal frequency corresponding to the maximum velocity is just the out-step frequency of the microswimmers.

**Figure 3:**
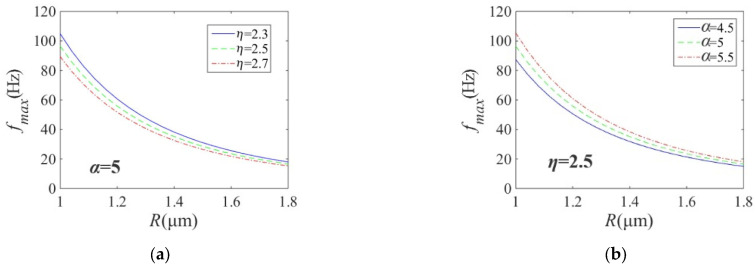

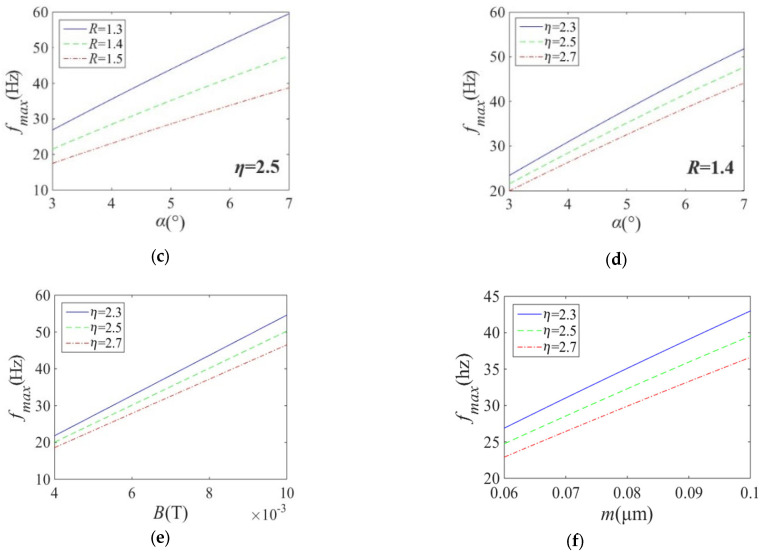
Out-step frequency and its changes. (**a**) radius *R* and viscosity *η* changes. (**b**) radius *R* and helix angle α changes. (**c**) helix angleαand radius *R* changes. (**d**) helix angleαand viscosity *η* changes. (**e**) *B* and *η* changes. (**f**) *m* and *η* changes.

Here, the out-step frequency of the microswimmers as functions of the related parameters is investigated (see Figure 3).

When the helix angle is constant (α = 5°), the out-step frequency of the microswimmers decreases with increasing helix radius *R* and fluid viscosity *η*. When the helix radius *R* increases, the effects of the fluid viscosity *η* on the out-step frequency of the microswimmers becomes small. When the fluid viscosity is constant (*η* = 2.5 mPa·s), the out-step frequency of the microswimmers decreases with increasing the helix radius *R* and decreasing the helix angle α. When the helix radius *R* increases, the effects of the helix angle α on the out-step frequency of the microswimmers becomes small. 

When the fluid viscosity is constant (*η* = 2.5 mPa·s), the out-step frequency of the microswimmers increases with decreases of the helix radius *R* and increases of the helix angle α. When the helix angle α increases, the effects of the helix radius *R* on the out-step frequency of the microswimmers becomes large. When the helix radius is constant (*R* = 1.4 μm), the out-step frequency of the microswimmers increases with decreasing fluid viscosity *η* and increasing helix angle α. When the helix angle α increases, the effects of the fluid viscosity *η* on the out-step frequency of the microswimmers becomes large. 

When other parameters are constant, the out-step frequency of the microswimmers increases with decreasing fluid viscosity *η* and increasing magnetic field intensity *B*. When the magnetic field intensity *B* increases, the effects of the fluid viscosity *η* on the out-step frequency of the microswimmers becomes large. When other parameters are constant, the out-step frequency of the microswimmers increases with decreasing fluid viscosity *η* and increasing thickness *m* of the magnetic layer. When the thickness *m* of the magnetic layer increases, the effects of the fluid viscosity *η* on the out-step frequency of the microswimmers becomes large. 

In order to illustrate the above analysis, driving experiments of microswimmers are conducted. The preparation process of a magnetically driven spiral micro-swimmer includes evaporative plating sacrificial layer, dispersing spiral carbon nanotubes, evaporating magnetic layer, and micro-robot stripping. The evaporation is conducted with a vacuum evaporation plating machine. Metal aluminum is used as a sacrificial layer and will enter the sample solution after corrosion. Here, isopropyl alcohol is selected for the dispersion of spiral carbon nanotubes. The magnetic layer on the surface of spiral carbon nanotubes is made of ferromagnetic material nickel. In order to tightly coat the magnetic layer on the surface of carbon nanotubes on their tiny spiral structure, the evaporation rate should be kept at a low speed. Here, the evaporation speed of 0.3 Å/s is used. According to the requirements in subsequent driving experiments, the magnetic layer thickness is selected to be 70 nm, 80 nm, 90 nm and 100 nm. 

**Figure 4:**
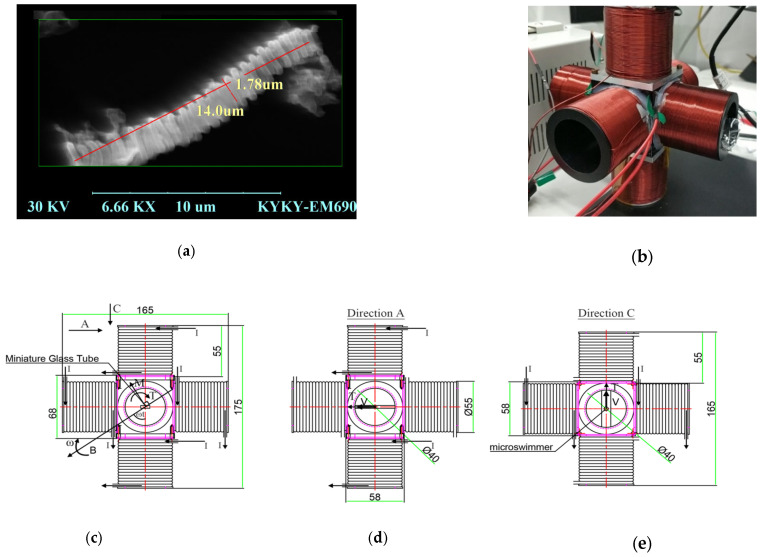
Micro spiral swimmers and driving coils. (**a**) microswimmer. (**b**) magnetic coils. (**c**) coil front view. (**d**) coil side view. (**e**) coil vertical view.

The effect of the magnetic layer thickness on the out-step frequency of microswimmers is measured. The micro-swimmers with magnetic layer thicknesses of 70 nm, 80 nm, 90 nm, and 100 nm are dispersed, respectively, and the sample solutions with a glycerol concentration of 30% are prepared, respectively. The sample solution is taken with capillary tube and fixed at the center of rotating magnetic field, and the magnetic field intensity is 8 mT (Figure 4a shows the microswimmer, and Figure 4b shows the developed electromagnetic coil). 

Figure 4c,d and e provide the CAD drawing showing dimensions of the setup and vector of B (external magnetic field) and M (magnetization of the robot). Here, a miniature glass tube is placed in the center of the setup and the micro swimmer is in the miniature glass tube. B is the external magnetic field and *M* is the magnetization of the robot. They are rotated about the center of the setup to produce a driving torque *T* for the rotation of the micro swimmer. Under the driving torque *T*, the micro swimmer moves within the miniature glass tube at the velocity *V*. 

**Figure 5:**
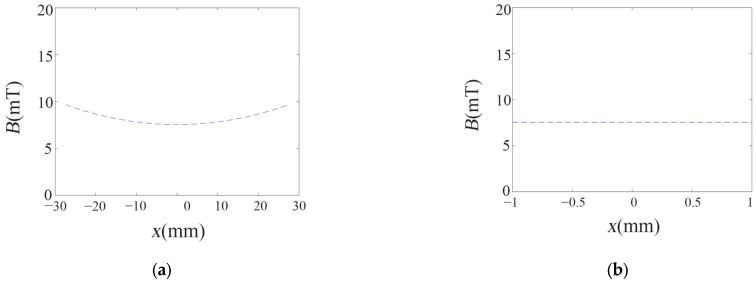
External magnetic field distribution in coils. (**a**) from −30 mm to 30 mm. (**b**) from −1 mm to 1 mm.

In the Helmholtz coil, the radius of the coil should be equal to the distance between the two same phase coils. In the coils in this paper, the structure is similar to the Helmholtz coil. However, there is a small difference between them. In the coils in this paper, the diameter of the coil should be equal to the distance between the two same phase coils. For the coils in this paper, the external magnetic field is simulated (see Figure 5). 

In the coils, the external magnetic field changes slowly in the axis direction of the coil. In the test, the miniature glass tube is placed on the center of the setup and the microswimmer moves in the range of 2 mm. Here, the external magnetic field is almost unchanged. The change of the external magnetic field is below 0.13%. So, it can be considered to be a uniform magnetic field without gradient.

**Figure 6:**
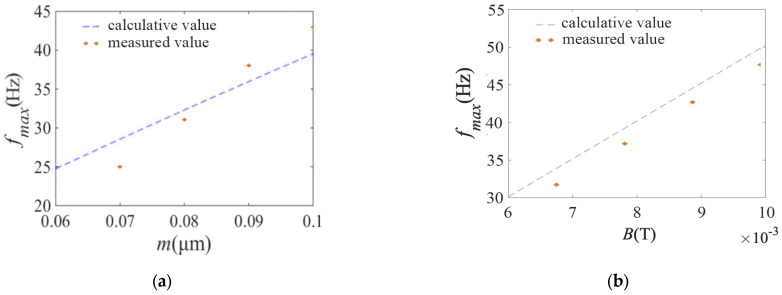
Driving experiments of micro spiral swimmers. (**a**) out-step frequency change with *m*. (**b**) out-step frequency change with *B*.

The DC power supply, the signal generator, the power amplifier, and the microscope are turned on one by one. The frequency of the signal output is at 0 Hz and gradually increases. The velocity of the microswimmer increases with the signal frequencies. When the signal frequency increases to a certain value, the velocity of the microswimmer begins to decrease. It is just the out-step frequency of the microswimmer. Out-of-step frequencies of the microswimmers under four different magnetic layer thicknesses are measured (see Figure 6a besides it, for the magnetic layer thickness of 70 nm), changing the magnetic field intensity, the out-of-step frequencies of the microswimmers under four different magnetic field intensities are measured (see Figure 6b). Results show that the experimental and theoretical analysis of the changes of the out-step frequency of the microswimmer with the thickness of the magnetic layer and magnetic field intensity have good consistency. The maximum error is 12.5% at 70 nm and 8 mT. the analysis is illustrated in this paper.

Compared with other similar results [28,29,30,31,32,33], the microswimmer studied in this paper are directly prepared using commercially available spiral carbon nanotubes, which have a simple process and low cost, thus avoiding the expensive preparation process of complex micro spiral structures. Compared with the microswimmer based on spiral water-conducting vessels of the plants in reference [18], the microswimmer studied here has a smaller size and more efficient spiral propulsion. In reference [18], the manufactured spiral microswimmer has a length of about 50 microns and a diameter of about 10 microns, with a speed of 250 μm/s, which responds to a relative speed of 5 body lengths/s. In this research, the manufactured spiral microswimmer has a length of about 14 microns and a diameter of about 1.8 microns, and with a speed of 130 μm/s, which responds to a relative speed of 9 body lengths/s.

## 5. Conclusions

A microswimmer with a magnetic layer on the surface of the spiral carbon nanotubes was proposed. Its mechanical model was proposed and the equations of the velocities and out-step frequency were deduced. Using these equations, the velocities and out-step frequency of the microswimmer and their changes with related parameters were investigated. Results showed that the velocities of the microswimmers were proportional to the radius and helix angle of the spiral carbon nanotubes. The out-step frequencies of the microswimmers were proportional to the magnetic field strength, the helix angle, and the magnetic layer thicknesses of the spiral carbon nanotubes. The out-step frequencies of the microswimmers were inversely proportional to the fluid viscosity. The microswimmers were produced and measured. The experimental results were in good agreement with the calculative ones. The microswimmer could be used to conduct targeted drug transportation via injecting into the human body, or they can be used for minimally invasive surgery to operate in the eyes, inner ear, and cardiovascular system.
